# AI based prediction of severe exacerbation in Asian bronchiectasis patients using the KMBARC registry

**DOI:** 10.1038/s41598-026-38968-9

**Published:** 2026-02-25

**Authors:** Bumhee Yang, Sun-Hyung Kim, Geun-Hyeong Kim, Geonhui Min, Inyoung Jang, Dong Eun Kye, Kyungsang Kim, Ji Ye Jung, Ju Ock Na, Seung Park, Hyun Lee

**Affiliations:** 1 Division of Pulmonary and Critical Care Medicine, Department of Medicine, Chungbuk National University Hospital, Chungbuk National University College of Medicine, 776, 1sunhwan-ro, Seowon-gu, Cheongju, 28644 Republic of Korea; 2 Center for Advanced Medical Computing and Analysis, Department of Radiology, Massachusetts General Hospital, Harvard Medical School, Boston, MA USA; 3 The Medical Artificial Intelligence Center, Chungbuk National University Hospital, Chungbuk National University College of Medicine, 776, 1sunhwan-ro, Seowon-gu, Cheongju, 28644 Republic of Korea; 4 Department of Biochemistry, Chungbuk National University College of Medicine, 1, Chungdae-ro, Seowon-gu, Cheongju, 28644 Republic of Korea; 5 Department of Medicine, Chungbuk National University College of Medicine, 1, Chungdae-ro, Seowon-gu, Cheongju, 28644 Republic of Korea; 6 Division of Pulmonary and Critical Care Medicine, Department of Internal Medicine, Severance Hospital, Yonsei University College of Medicine, 50-1, Yonsei-ro, Seodaemun-gu, Seoul, 03722 Republic of Korea; 7 Division of Pulmonology, Department of Internal Medicine, Soonchunhyang University, College of Medicine, 44, soonchunhyang 4-gil, Dongnam-gu, Cheonan, 31151 Korea; 8 Division of Pulmonary Medicine and Allergy, Department of Internal Medicine, Hanyang University College of Medicine, 222-1, Wangsimni-ro, Seongdong-gu, Seoul, 04763 Republic of Korea

**Keywords:** Artificial intelligence, Acute exacerbation, Bronchiectasis, Prediction, Respiratory tract diseases, Machine learning

## Abstract

Classical scoring tools such as the Bronchiectasis Severity Index (BSI) and FACED have shown good performance for predicting adverse outcomes, yet they were developed using European cohorts and may not fully align with Asian clinical characteristics. This study aimed to develop an Asian population–specific artificial intelligence (AI) model for predicting severe acute exacerbations (AEs) in bronchiectasis. A total of 492 patients with 1-year follow-up data from the Korean Multicenter Bronchiectasis Audit and Research Collaboration registry were analyzed. Severe AE was defined as an event requiring an emergency department visit or hospitalization due to worsening respiratory symptoms. Three AI models—extreme gradient boosting, logistic regression, and multilayer perceptron (MLP)—were trained and compared with classical scoring models. Severe AE occurred in 56 patients (11.4%). Among the models, the MLP demonstrated the best performance, with higher sensitivity, specificity, F1-score, and area under the receiver operating characteristic curve compared with both classical scores. Shapley additive explanation (SHAP) analysis identified BSI, sputum characteristics, and histories of tuberculosis and pneumonia as key predictors. Although classical scores performed well, a population-specific AI model using local clinical data showed improved predictive performance and may support individualized risk assessment for Asian patients with bronchiectasis.

**Supplementary Information:**

The online version contains supplementary material available at 10.1038/s41598-026-38968-9.

## Introduction

Individuals with bronchiectasis experience chronic respiratory symptoms and recurrent infections, often leading to worsening symptoms known as acute exacerbations (AEs)^1^. Severe AEs, requiring emergency room visits or hospitalization, are especially concerning due to their association with increased morbidity and mortality. These events necessitate more intensive medical interventions and extended hospital stays^2–4^. Therefore, predicting severe AEs in patients with bronchiectasis is crucial to improving clinical outcomes and effectively managing healthcare resources.

Classical scoring models such as the Bronchiectasis Severity Index (BSI) and FACED have been widely used to assess the risk of severe AE in patients with bronchiectasis^2,3^. While these models have provided valuable insights, they were originally developed to predict long-term mortality and may not be optimized for predicting severe AE. Additionally, they may not fully consider individual patient characteristics or comorbidities, which may limit their predictive performance. Another limitation is that these models assigned weights to clinical indicators in a linear manner. Specifically, they may not fully account for how variables interact in nonlinear or dynamic ways, such as synergistic effects where combined factors have a greater impact than each factor alone or changes in relationships over time. Furthermore, since classical scoring models were developed using cohorts of European ancestry, their applicability to Asian populations may require consideration of additional clinical factors such as a history of tuberculosis (TB).

**Fig. 1:**
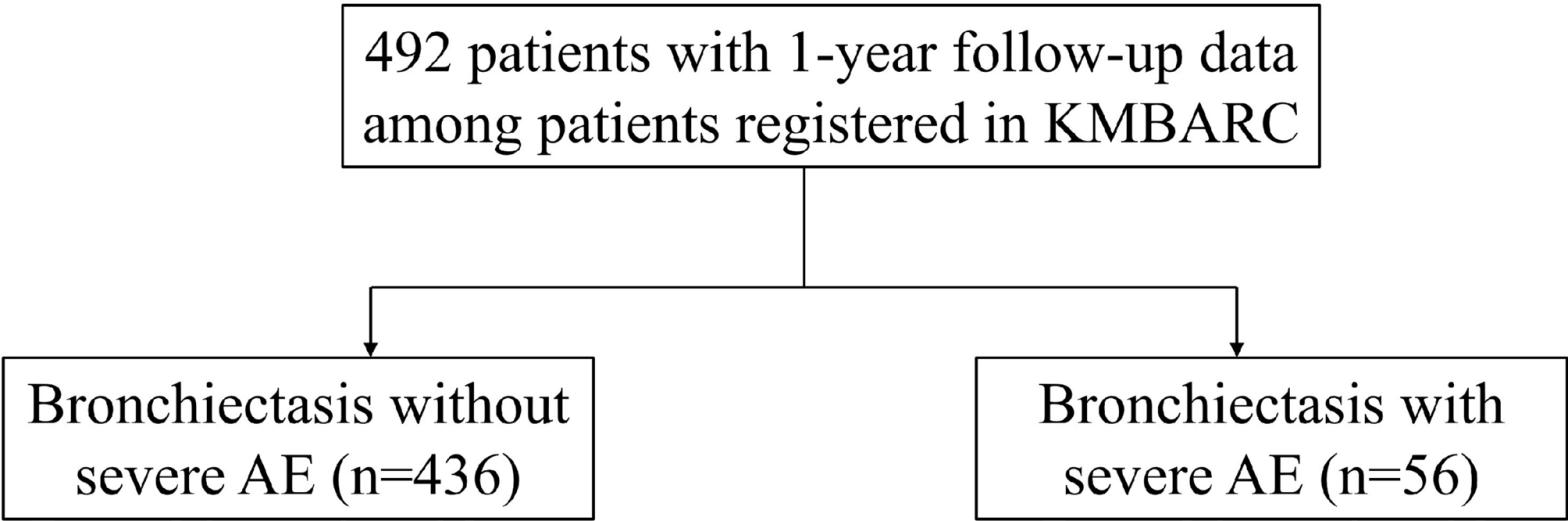
Study population. Abbreviations: KMBARC, Korean Multicenter Bronchiectasis Audit and Research Collaboration; AE, acute exacerbation.

Recently, there has been an increasing application of artificial intelligence (AI) models in the medical field, effectively analyzing complex clinical data and predicting disease outcomes^5,6^. However, in the field of bronchiectasis, as far as we know, no studies have attempted to develop AI models to predict outcomes. Since AI models can be tailored to population-specific data, there is a potential to create a model that more accurately predicts severe AE in Asians compared to classical scoring models based on European ancestry. Therefore, this study aimed to develop an AI model specific to the Asian population to predict severe AE (Fig. 1).

## Results

### Baseline characteristics

The baseline characteristics of the 492 patients with bronchiectasis are presented in Table 1. The most common comorbidity was chronic obstructive pulmonary disease (43·9%), followed by history of pulmonary tuberculosis (35·6%) and asthma (25·4%). The most commonly identified sputum was mucoid (63·8%), followed by mucopurulent (29·6%). *Pseudomonas aeruginosa* was identified in 22% of patients with bronchiectasis. Among the respiratory treatments, mucolytics (70%) were the most frequently used, followed by long-acting muscarinic antagonists (20·9%) and combination therapies of long-acting beta_2_ agonists/long-acting muscarinic antagonists (18·2%).

**Table 1:** Baseline Characteristics.

Age, years	*N* = 492	Without severe AE (*n* = 436)	With severe AE (*n* = 56)	*P*-value
	64·5 (55·5–73·5)	64·7 (55·6–73·7)	63·5 (55·6–71·5)	0·3600
Sex, females	259 (52·6)	225 (51·6)	34 (60·7)	0·2530
BMI, kg/m^2^	23·1 (19·5–26·7)	23·2 (19·7–26·8)	22·0 (18·0–26·0)	0·0176
Smoking history				
Never smoker	312 (63·4)	277 (63·7)	35 (62·5)	0·9703
Ex-smoker	160 (32·5)	141 (32·4)	19 (33·9)	
Current smoker	19 (3·9)	17 (3·9)	2 (3·6)	
Comorbidities				
Chronic obstructive pulmonary disease	216 (43·9)	184 (42·2)	32 (57·1)	0·0479
Asthma	125 (25·4)	104 (23·9)	21 (37·5)	0·0408
History of pulmonary tuberculosis	174 (35·6)	148 (34·2)	26 (46·4)	0·0983
Diabetes mellitus	55 (11·2)	48 (11·0)	7 (12·7)	0·8777
Stroke	9 (2·3)	9 (2·6)	0 (0·0)	0·6069
Pertussis	44 (9·0)	41 (9·5)	3 (5·4)	0·4451
NTM-PD	48 (9·8)	41 (9·5)	7 (12·5)	0·6321
Rheumatoid arthritis	32 (6·5)	29 (6·7)	3 (5·4)	1·0000
Sinusitis	42 (8·5)	39 (8·9)	3 (5·4)	0·4560
Gastroesophageal reflux disease	71 (14·5)	61 (14·1)	10 (17·9)	0·5810
History of pneumonia	209 (42·7)	167 (41·1)	42 (50·6)	0·142
Spirometry				
FVC, L	2·6 (1·8–3·4)	2·6 (1·8–3·4)	2·1 (1·4–2·9)	< 0·001
FVC, %	71·9 (55·8–88·0)	73·2 (57·9–88·5)	61·4 (43·6–79·2)	< 0·001
FEV1, L	1·7 (1·1–2·3)	1·7 (1·1–2·3)	1·3 (0·7–1·8)	< 0·001
FEV1, %	63 (44–82)	64·2 (45·6–68·0)	48·8 (29·6–68·0)	< 0·001
FEV1/FVC, %	65 (51–79)	65·6 (52·0–79·1)	60·1 (46·7–73·5)	0·0049
Sputum color				
Mucoid	293 (63·8)	273 (67·2)	20 (37·7)	< 0·001
Mucopurulent	136 (29·6)	114 (28·1)	22 (41·5)	
Purulent	26 (5·7)	17 (4·2)	9 (17·0)	
Severe purulent	4 (0·9)	2 (0·5)	2 (3·8)	
Sputum volume	10.0 (5.0-25.0)	10.0 (5.0-25.0)	25.0 (10.0-100.0)	< 0·001
mMRC	1 (0–2)	1·1 (0·3–1·8)	1·8 (0·7–2·9)	< 0·001
BHQ score	64·2 (53·6–74·6)	64·7 (54·5–74·9)	61.5 (50·6–72·4)	< 0·009
Modified Reiff score	6 (2–10)	6·1 (2·1–10·1)	8·8 (4·5–13·1)	< 0·001
Microbiology				
P. aeruginosa	108 (22)	81 (18·6)	27 (48·2)	< 0·001
BSI score	6·1 (2·7–9·5)	5·2 (2·9–7·6)	12·8 (9·8–15·9)	< 0·001
FACED score	2·0 (0·4–3·6)	1·9 (0·4–3·5)	3·09 (1·7–4·5)	< 0·001
Previous history of AE	105 (21.3)	75 (17.2)	30 (53.6)	< 0·001
Laboratory findings				
WBC count, /L	7300 (4800–9800)	7160 (4750–9570)	8430 (5830–11030)	0·0051
Neutrophil count, /µL	4378 (2274–6482)	4253 (2272–6234)	5416 (2716–8117)	0·0236
Eosinophil count, /µL	196 (26–366)	195 (26–363)	204 (25–383)	0·7550
Hemoglobin	13.0 (12.0–14.0)	13·3 (11·9–14·6)	12·6 (11·3–13·8)	0·0111
Platelet count	243 (164–321)	237 (162–312)	290 (196–384)	0·0012
hs-CRP, mg/dL	0.42 (0.15-1.10)	0.40 (0.14-1.10)	0.75 (0.38-0.96)	0·2297
Respiratory treatment	398 (81·9)			
Long-term oxygen therapy	17 (3·5)	6 (1·4)	11 (20·0)	< 0·001
Theophylline	41 (10·1)	30 (8·5)	11 (22·0)	0·0065
ICS	16 (5·1)	10 (3·6)	6 (17·7)	0·0036
ICS + LABA	11 (3·7)	10 (3·7)	1 (3·7)	1·0000
LABA + LAMA	55 (18·2)	49 (17·8)	6 (21·4)	0·8299
LAMA	68 (20·9)	58 (19·7)	10 (31·3)	0·1955
Oral corticosteroid	10 (3·3)	7 (2·6)	3 (10·3)	0·0614
Mucolytics	285 (70·0)	250 (69·6)	35 (72·9)	0·7657
Macrolide	36 (7·7)	28 (6·7)	8 (15·4)	0·0466

Data are presented as medians (interquartile ranges) or numbers (%).

Abbreviations: AE, acute exacerbation; BMI, body mass index; NTM-PD, Non-tuberculous mycobacterial pulmonary disease; FVC, forced vital capacity; FEV1, forced expiratory volume in 1 s; mMRC, *modified Medical Research Council*; BHQ, The Bronchiectasis Health Questionnaire; P. aeruginosa, *Pseudomonas aeruginosa*; BSI, *Bronchiectasis* Severity Index; FACED FEV1% predicted (F), age (A), chronic colonization by Pseudomonas aeruginosa (C), radiological extent of the disease (E), and dyspnea (D); WBC, White blood cell; hs-CRP, high-sensitivity C-reactive protein; ICS, inhaled corticosteroid; LABA, long-acting beta-2 agonist; LAMA, long-acting muscarinic antagonist.

### Quantitative evaluation

**Fig. 2:**
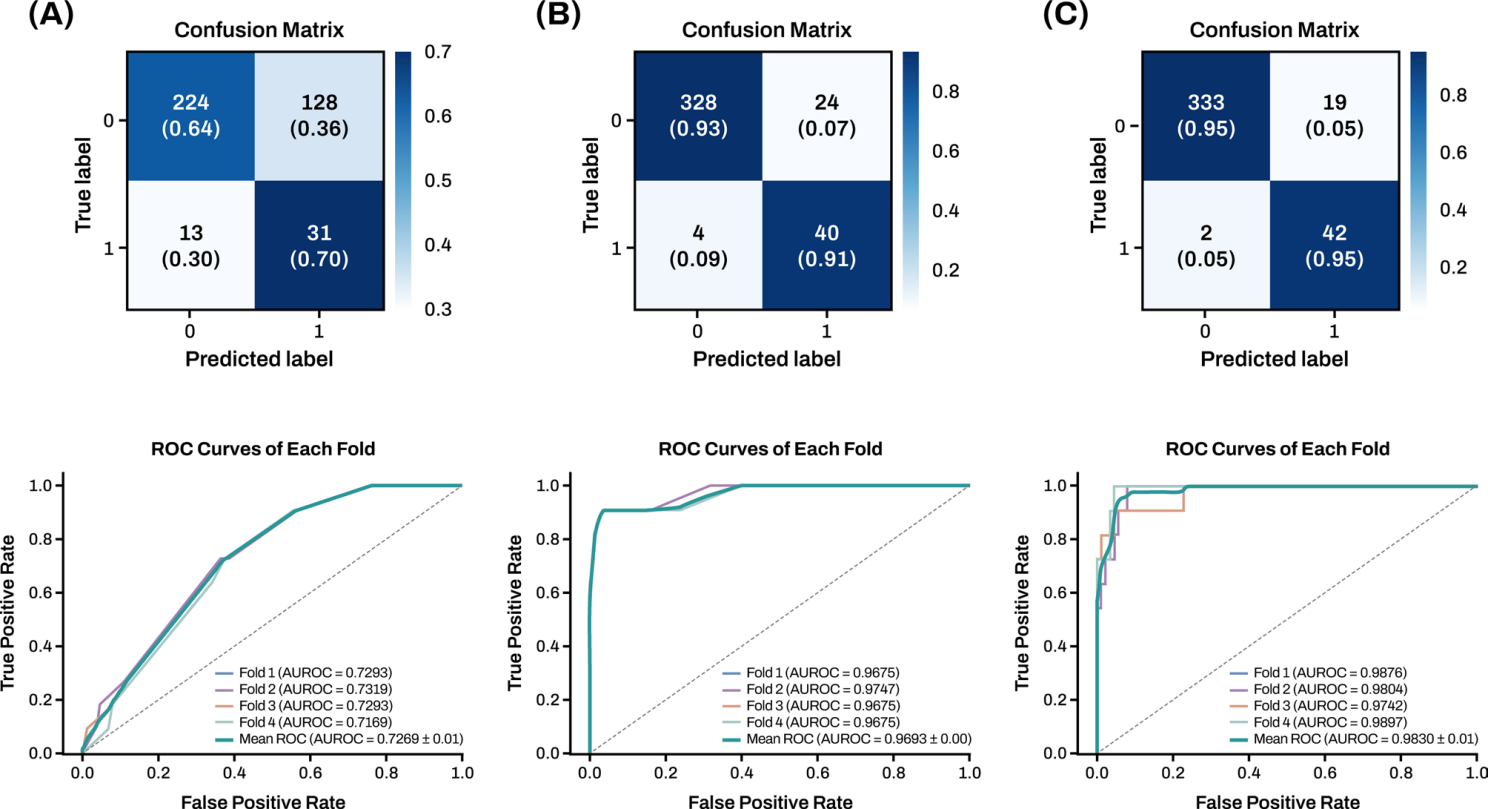
Confusion matrix sum (top) and Receiver Operating Characteristic (ROC) curve plot (bottom) for the MLP model evaluated through four-fold cross-validation. Input features: (**a**) Bronchiectasis Severity Index (BSI), (**b**) FACED score, and (**c**) all features combined. Abbreviations: MLP, multilayer perceptron; FACED score,FEV1% predicted (F), age (A), chronic colonization by Pseudomonas aeruginosa (C), radiological extent of the disease (E), and dyspnea (D); AUROC, Area Under the Receiver Operating Characteristic Curve.

As presented in Fig. 2, we implemented a stratified four-fold cross-validation procedure to ensure robust performance evaluation while preserving the proportion of patients with severe acute exacerbation (AE) across all folds. Among the 492 patients in the KMBARC registry, 56 patients (approximately 11.4%) experienced severe AE. To mitigate sampling bias and ensure consistent distribution of the minority class (severe AE), each fold was carefully constructed to maintain a similar ratio of AE cases, with stratified sampling based on the outcome label. In each iteration, three folds were used for training and one for validation, and the process was repeated across four permutations. An independent 20% test set was held out and used in each cycle to evaluate final model performance.

Given the class imbalance between exacerbators and non-exacerbators, we adopted several measures to mitigate potential bias. First, we emphasized evaluation metrics that are less sensitive to imbalance, such as the area under the receiver operating characteristic curve (AUROC) and the F1-score, rather than relying on overall accuracy. Second, within the MLP architecture, dropout regularization (dropout rate = 0.2) and batch normalization layers were applied to prevent overfitting, which could be exacerbated by imbalance. Lastly, the SHAP (Shapley Additive Explanations) analysis was used not only to enhance model interpretability, but also to confirm that the model was not disproportionately influenced by dominant features alone.

With BSI as the input (Fig. 2a), the model achieved an average sensitivity of 0·90, specificity of 0·93, F1-score of 0·74, and AUROC of 0·97. In comparison, using all baseline clinical, radiologic, microbiologic, and functional features summarized in Table 1 (Fig. 2c), these metrics improved to 0·95, 0·95, 0·80, and 0·98, respectively.

### Performance comparison

To better predict severe AE, we compared its performance with those of classical scoring models and models (Table 3). For classical scoring models, severe AE prediction was measured using the BSI and FACED scores. The predictive rate of severe AE using the BSI and FACED score was as follows: sensitivity (0·91 vs. 0·73), specificity (0·93 vs. 0·63), F1-score (0·74 vs. 0·31) and AUROC (0·97 vs. 0·73).

**Table 2:** Predictive performance for various clinical indicators and models.

Classifier		Sensitivity	Specificity	F1-score	AUROC
Clinical indicator	FACED	0·73	0·63	0·31	0·73
	BSI	0·91	0·93	0·74	0·97
Model	XGBoost	0·91	0·92	0·73	0·96
	LR	0·91	0·92	0·72	0·97
	MLP	0·95	0·95	0·80	0·98

Abbreviations: FACED: FEV1% predicted (F), age (A), chronic colonization by *Pseudomonas aeruginosa* (C), radiological extent of the disease (E), and dyspnea (D); BSI, Bronchiectasis Severity Index; XGBoost, extreme gradient boosting; LR, logistic regression; MLP, multilayer perceptron; AUROC, Area Under the Receiver Operating Characteristic Curve.

**Fig. 3:**
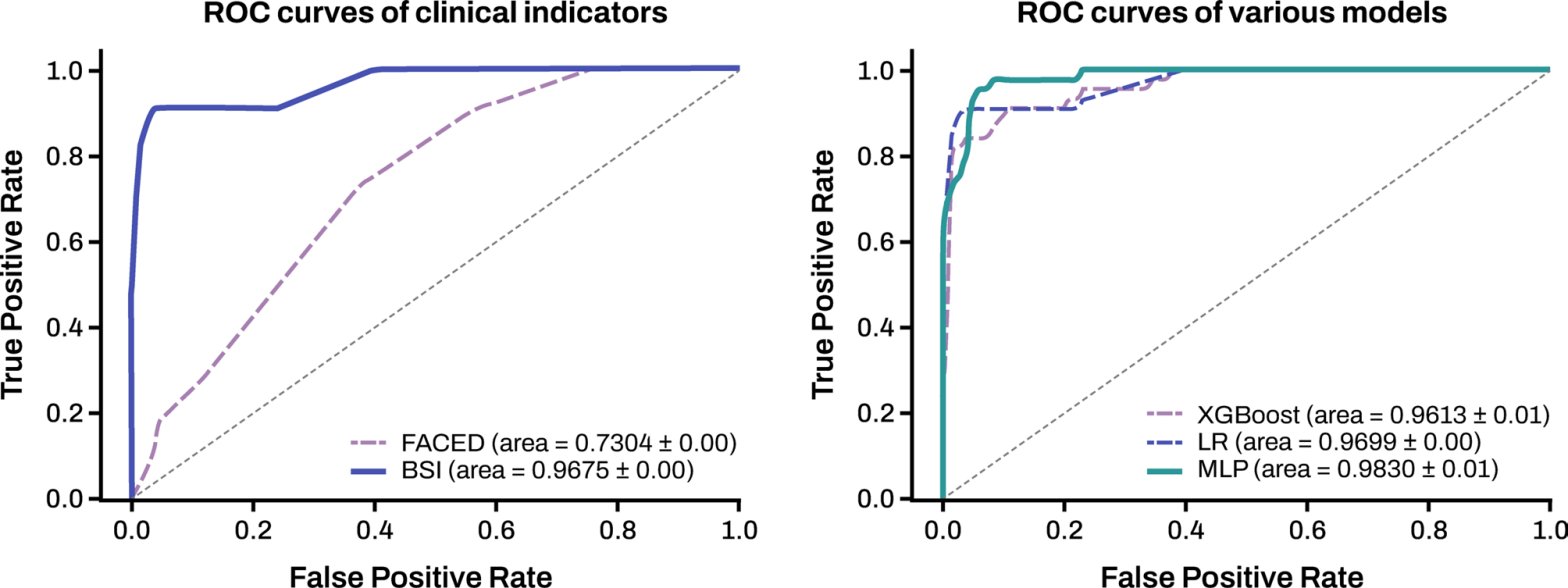
ROC curves of clinical indicators and various models. Abbreviations: XGBoost, Extreme gradient boosting; LR, logistic regression; MLP, multilayer perceptron.

The classical scoring model predictions were compared with those of the three analysis models (XGBoost, LR, and MLP). The MLP model showed the best performance in terms of sensitivity (0·95), specificity (0·95), F1-score (0·80), and AUROC (0·98). The receiver operating characteristic (ROC) curves for the three predictive models are shown in Fig. 3.

### Visualization of factors affecting prediction

**Fig. 4:**
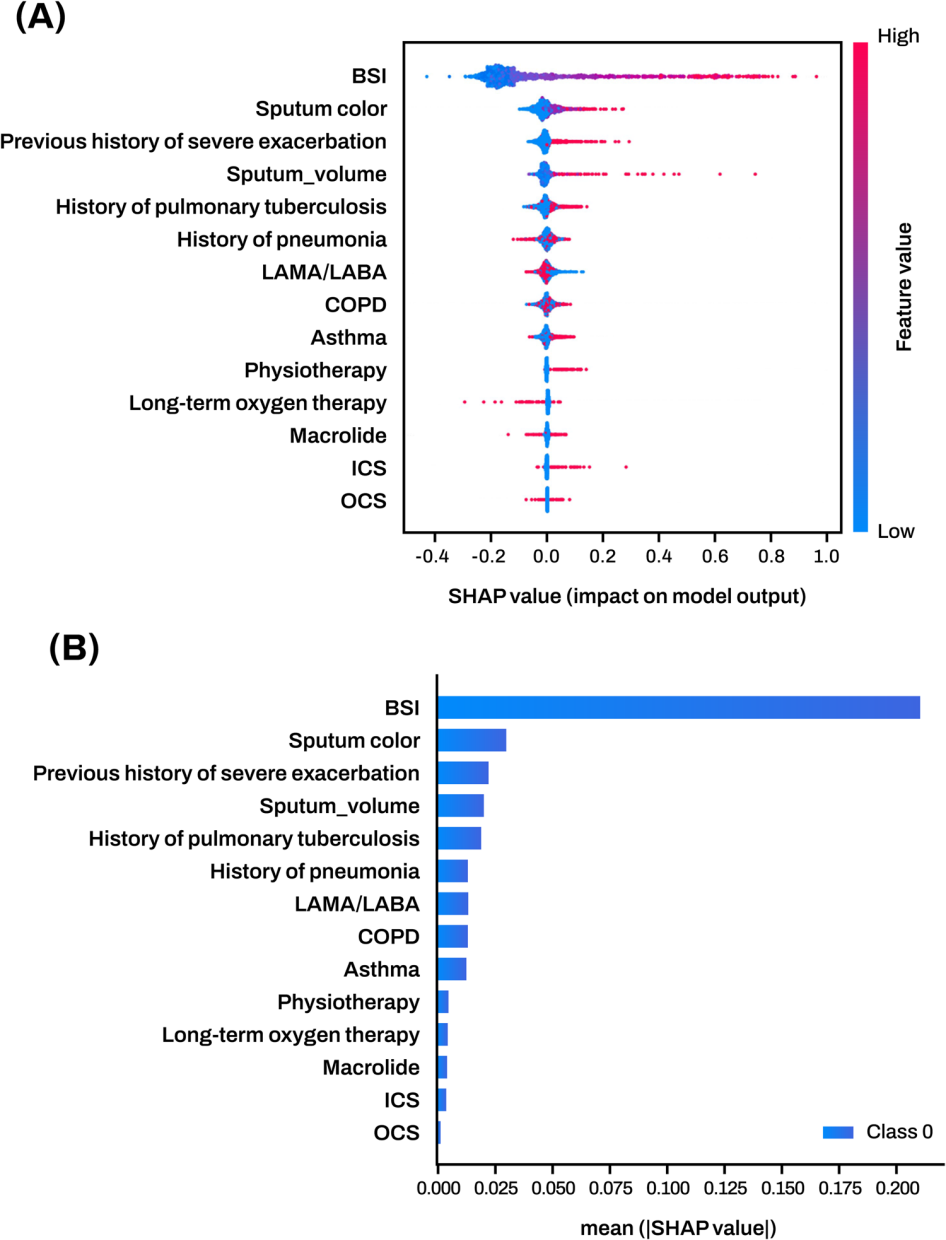
**(A**) Summary plots of numeric data contributions. This figure contains the entire numeric data used to develop the MLP model. The magnitude is indicated by the colored line on the right side. The horizontal axis represents the SHAP values for each feature, and the vertical axis represents the most (top) to least (bottom) influential features in the MLP model. (**B**) Absolute summary plot illustrated by averaging the absolute SHAP values for the numerical data. Abbreviations: SHAP, Shapley Additive Explanations; BSI, Bronchiectasis Severity Index; LAMA, long-acting muscarinic antagonist; LABA, long-acting beta-2 agonist; COPD, Chronic Obstructive Pulmonary Disease; ICS, inhaled corticosteroid; OCS, Oral corticosteroid.

We utilized the Shapley Additive Explanation (SHAP) method to explore how the input features contributed to the output of the MLP model. SHAP calculates the relative importance of each feature with respect to the prediction outcome. The magnitude of the SHAP value reflects the extent of the influence of each feature on the prediction A positive SHAP value suggests that the feature increases the probability of severe AE in patients with bronchiectasis, whereas a negative SHAP value indicates that the feature reduces the probability. For instance, a negative SHAP value for BSI means that a lower BSI score contributes to a lower predicted risk, which aligns with its clinical interpretation. SHAP value indicates that the feature reduces the probability. Figure 4A presents a SHAP summary plot for all continuous numerical features used in the MLP model for prediction, including BSI, sputum volume, pulmonary function metrics, and other scalar variables. Among the numeric data, the most influential features in the MLP model were BSI, increased sputum volume and color, previous severe AE history, and history of tuberculosis and pneumonia, in that order. Figure 4B provides a bar plot of the mean absolute SHAP values for the same numerical features shown in Fig. 4A.

To enable comparison of feature importance across different modeling approaches, we further applied SHAP analysis to the XGBoost and logistic regression (LR) models. While the MLP model’s SHAP results are presented in Supplemental Fig. 3, SHAP summary plots for XGBoost and LR are provided in Supplemental Fig. 3. Although the exact magnitude and ranking of SHAP values varied, the consistency in the selected features supports the robustness of the predictors identified. These results suggest that clinically relevant factors are preserved across distinct machine learning architectures, providing additional confidence in the model generalizability.

## Discussion

This study is the first to develop a population-tailored AI model that utilizes longitudinal data from the KMBARC to predict severe AE in Korean patients with bronchiectasis. A key strength lies in the encouraging performance of the MLP AI model in predicting severe AE in the Korean population, showing potential improvement compared with the widely used classical scoring systems (BSI and FACED scores) in terms of sensitivity, specificity, and overall predictive accuracy. Using the SHAP model, we identified factors such as sputum characteristics and tuberculosis history that help explain how AI achieves more accurate severe AE predictions.

Classical scoring models, such as the BSI and FACED, have been instrumental in identifying patients at high risk of mortality and AE. It should be noted that both the FACED and BSI scores were originally developed for predicting long-term mortality rather than short-term exacerbations^2,3^. Nevertheless, because severe AE is known to negatively affect long-term prognosis, predicting AE may provide indirect insights into mortality risk and help guide individualized management^7,8^. However, these models assign weights to specific clinical indicators in a linear manner, which limits their ability to capture the complex and nonlinear interactions between variables. For instance, the BSI and FACED do not account for potential synergistic effects or dynamic relationships among clinical features, which can reduce accuracy when predicting short-term outcomes such as AE. Machine learning (ML) models, including the MLP, address these limitations by employing nonlinear learning techniques that allow the identification of intricate patterns in data. The MLP model, with its multiple layers and nonlinear activation functions, appeared to achieve higher predictive performance by considering a wide range of factors that influence severe AE^9–11^. Building on this, the SHAP analysis in our study provided insights into the complex interactions and relative contributions of these variables, emphasizing their combined influence on prediction accuracy.

In this study, the interpretability of the MLP model was implemented using SHAP analysis, which provided insights into key predictors and their interactions. When the AI model incorporated sputum characteristics and histories of tuberculosis and pneumonia in addition to the BSI, it showed improved ability to predict severe AE. Specifically, SHAP interaction analysis revealed that the combination of a history of tuberculosis—a factor prevalent in Asian populations—and purulent sputum characteristics had a synergistic effect on increasing the risk of severe AE, rather than a simple additive effect. While the linear BSI model might underestimate the risk in these patients if their overall score is moderate, the MLP model successfully captured this nonlinear amplification of risk, contributing to its superior sensitivity and AUROC. These findings support the potential role of AI in developing population-specific prediction tools, highlighting that incorporating population-specific data enhances predictive performance. For example, in our study, by considering tuberculosis history—common in Asian patients with bronchiectasis but not in European patients—AI could develop an individualized prediction model. This approach could serve as a foundation for future studies in other Asian countries aimed at developing and validating population-specific AI models for bronchiectasis.

Despite its strengths, this study has several limitations. First, the study population was drawn from referral centers, where patients are typically managed by pulmonologists and may not represent community-based populations. This referral bias could limit the model’s generalizability to patients with different disease severities or lower adherence to care. Second, although internal validation was performed by dividing the study population and cross-checking the model’s validity, external validation was not conducted, which further restricts the generalizability of the findings. Third, despite inclusion of patients from tertiary hospitals, the number of severe AE events was relatively low (56/492, 11.4%), resulting in class imbalance between exacerbators and non-exacerbators. This imbalance could predispose the ML models to overfitting^12,13^, although the use of the AUROC curve as a quantitative performance indicator likely mitigated this issue. Future studies should incorporate external validation and explore advanced ML techniques, such as ensemble learning or deep learning architectures, to further improve predictive performance. Additionally, incorporating real-time data inputs, such as metrics from wearable technology, could enable dynamic updates to prediction models, allowing more precise and timely predictions. By refining these models and broadening their applicability, future research has the potential to advance personalized approaches that improve outcomes for patients with bronchiectasis.

## Conclusion

Although classical scoring models have shown good performance in predicting severe AE in Korean patients with bronchiectasis, our findings indicate that an MLP-based AI model offers additional predictive value. Nevertheless, the clinical implications remain to be established, and these preliminary findings from a single-country cohort require external validation before clinical application.

## Methods

### Study population

The present study included 492 patients with 1-year follow-up data registered in The Korean Multicenter Bronchiectasis Audit and Research Collaboration (KMBARC). The KMBARC is a prospective, non-interventional, observational cohort study of non-cystic fibrosis bronchiectasis in Korea. Patients aged 18 years or older with bronchiectasis in one or more lobes on chest CT were included. Patients with cystic bronchiectasis, traction bronchiectasis associated with interstitial lung disease (ILD), and pregnant patients were excluded^14,15^. Of the 492 patients, 56 patients experienced severe AEs and were included in further analysis (Fig. 1).

### Ethic declarations

The study protocol was reviewed and approved by the Institutional Review Board of Chungbuk National University Hospital (IRB No 2024-012-005). Patient information was anonymized and deidentified before the analysis. Therefore, the need for informed consent was waived. This study was conducted in accordance with the principles of the Declaration of Helsinki.

### Data collection

At the time of registry enrollment, all patients had stable bronchiectasis. The baseline data included age, sex, body mass index (weight in kilograms divided by height in meters squared), smoking history, comorbidities, medication history, and laboratory findings. Patient symptoms were evaluated using the modified Medical Research Council (mMRC) and validated using the Korean version of The Bronchiectasis Health Questionnaire (BHQ) for quality of life^16–18^. The pulmonary function tests were performed at the time of enrollment. Prebronchodilator and postbronchodilator spirometry were performed according to the American Thoracic Society/European Respiratory Society criteria^19^. The absolute values of forced expiratory volume in one second (FEV_1_) and forced vital capacity (FVC) were recorded. Additionally, the percentages of the predicted values for FEV_1_ and FVC were calculated using an automatic calculator with a reference equation obtained from a representative Korean sample^20^. Sputum volume (mL/day) and color were evaluated in patients with bronchiectasis. Sputum volume was estimated as 50mL using a small glass and 200mL using a cup. The sputum color was evaluated using a validated photographic sputum color chart. Sputum color was classified into four types: mucoid, mucopurulent, purulent, or severely purulent^21^. The sputum specimens were subjected to microbiological analysis according to standard methods^22^. Conventional semi-qualitative bacterial cultures were used. All samples underwent initial Gram staining prior to sputum culture, provided the Murray and Washington criteria were met^23^. Nontuberculous mycobacteria (NTM) lung disease was diagnosed using the microbiological criteria provided by the American Thoracic Society and Infectious Disease Society of America^24^. Radiographic severity of bronchiectasis was analyzed using the modified Reiff score^25^. The FACED (forced expiratory volume in 1 s [FEV_1_] %predicted, age, chronic colonization, extension, and dyspnea) and BSI scores were used to assess the clinical status and severity of bronchiectasis, as previously described^2,3^.

### Definition of severe AE

Severe AE of bronchiectasis was defined as a visit to the ED or hospital admission due to respiratory symptoms. Respiratory symptoms included coughing, changes in sputum volume/viscosity, sputum suppuration, dyspnea, exercise ability, fatigue, malaise, and hemoptysis, which lasted for more than 48 hours^26^.

### Feature engineering

Feature engineering plays a vital role in machine learning by enabling the extraction of valuable insights from raw datasets. The input features for all three AI-based models were derived from baseline patient characteristics summarized in Table 1. These included demographic variables (age, sex, body mass index), clinical history (e.g., smoking status, history of pulmonary tuberculosis or pneumonia, comorbidities such as COPD and asthma), sputum characteristics (color and volume), microbiological data (e.g., Pseudomonas aeruginosa colonization), medication use (e.g., mucolytics, bronchodilators, corticosteroids), pulmonary function test results (FEV₁, FVC), radiological severity assessed using the modified Reiff score, and composite clinical severity indices, including the Bronchiectasis Severity Index (BSI) and FACED score. Additionally, patient-reported outcome measures, including the modified Medical Research Council (mMRC) dyspnea scale and the Bronchiectasis Health Questionnaire (BHQ), were incorporated.

For feature engineering, we first considered the missing values within our dataset. Since our dataset had between 3 (1%) and 192 (39%) missing values per feature, we replaced the missing values according to the data characteristics as follows: mean (BSI, sputum volume and color) and mode (history of pulmonary tuberculosis and pneumonia, long-term oxygen therapy, physiotherapy, macrolide, long-acting muscarinic antagonist/long-acting beta agonist inhalers [LAMA/LABA], inhaled corticosteroids [ICS], oral corticosteroid [OCS]). Second, since sputum color can be mucoid, mucopurulent, purulent, or severely purulent, we applied label encoding according to the severity of the sputum condition in sputum color for data analysis. Additionally, data scaling was conducted to ensure that the features are consistent and avoid the influence of larger features on the analysis results. Therefore, we standardized the numerical data as follows:

$$\:\stackrel{\sim}{\mathrm{x}}=\:\frac{\mathrm{x}-{\upmu\:}}{{\upsigma\:}}$$

where $\:\mathrm{x}$, $\:{\upmu\:}$, and $\:{\upsigma\:}$ correspond to original data, mean value, and standard deviation, respectively.

### AI models predicting severe AE

We predicted severe AE using various AI models, including Extreme Gradient Boosting (XGBoost), Logistic Regression (LR), and Multilayer Perceptron (MLP). Each model possesses unique characteristics and offers distinct advantages for capturing patterns and making predictions. The subsequent subsections provide a detailed description of each model, and their hyperparameters are listed in Table 2. Note that each model was implemented using Scikit-learn 1.1.2 and Tensor Flow 2.4.1 library in Python 3.8 and was trained using the CUDA 11.0.3 toolkit on a desktop computer with an Intel Core i7-12700 @2.10 GHz Central Processing Unit and NVIDIA GeForce RTX 3090 Ti Graphics Processing Unit.

**Table 3:** Hyperparameters of each model used for severe AE prediction.

Model	Hyperparameters	values
XGBoost	n_estimators	100
	reg_alpha	0·1
	reg_lambda	0·1
	subsample	1·0
	colsample_bytree	0·8
	gamma	1
	learning_rate	0·1
	max_depth	3
	min_child_weight	1
LR	C	0·1
	Penalty	L1
MLP	Number of hidden nodes	[32, 64, 128]
	Dropout rate	0·2
	Optimizer	Adam
	Learning rate	1.0e-3
	Batch size	8
	Loss function	Weighted binary cross-entropy

Abbreviations: XGBoost, extreme gradient boosting; LR, logistic regression; MLP, multilayer perceptron.

### Extreme gradient boosting, XGBoost

XGBoost stands for “Extreme Gradient Boosting,” a popular ensemble learning algorithm that belongs to the gradient boosting family of machine learning techniques. It offers a flexible implementation where decision tree concepts are fully utilized, achieving high predictive performance by sequentially training an ensemble of decision trees and effectively combining their predictions. Moreover, XGBoost exhibits significantly faster execution times than do commonly employed algorithms such as Adaboost^27^.

### Logistic regression, LR

Logistic Regression is a supervised machine learning algorithm developed for learning classification problems. It is based on the concept of fitting a sigmoid function to data to estimate the probability of an instance belonging to a particular class. The sigmoid function, also known as the logistic function, maps values in a range of 0 to 1, making it suitable for probability estimation. In addition, in LR the coefficients associated with each predictor variable represent the change in the log-odds of the target class for a unit change in the corresponding predictor, assuming that all other variables are held constant. These coefficients can provide insights into the direction and magnitude of each predictor’s influence on the outcomes.

### Multilayer perceptron, MLP

The MLP is a widely used artificial neural network architecture that excels in solving various machine learning tasks. It consists of multiple layers of interconnected neurons, organized in a feedforward manner. Additionally, by applying weighted sums and activation functions, the MLP can capture complex nonlinear relationships between input features and output predictions. Through back-propagation and gradient descent, the MLP is trained to minimize the loss function by adjusting their weights and biases.

As shown Supplemental Fig. 1, MLP developed in this study to predict severe AE consists of three MLP blocks and one output layer. Specifically, each MLP block comprises a dense layer, batch normalization layer, rectified linear unit (ReLU) activation, and dropout layer. The dropout rate was set to 0.2 to avoid overfitting. For the output layer, a dense layer followed by sigmoid activation was used to calculate the probability of severe AE.

### Quantitative evaluation

To evaluate the performance of each model, we used 20% of our dataset as the test dataset and performed four evaluations through four-fold cross-validation, which effectively controlled model overfitting. Specifically, the dataset excluding the test dataset was divided equally into four folds, where the number of severe AE cases was evenly distributed. Subsequently, four validation iterations were performed; in each iteration, one fold was used for validation and the remaining folds were used for training. The test dataset was used for actual model evaluation at each iteration.

In addition, we adopted four quantitative model performance metrics: sensitivity, specificity, F-score, and area under the receiver operating characteristic curve (AUROC), where the ROC curve is produced by plotting sensitivity on the y-axis against 1–specificity. In this study, the F-score refers specifically to the F1-score, defined as the harmonic mean of precision and sensitivity. The evaluation metrics were calculated using the following formulas:

Sensitivity = TP/(TP + FN).

Specificity = TN/(TN + FP).

F1-score = 2×(Precision×Sensitivity)/(Precision+Sensitivity).

Precision = TP/(TP + FP).

where TP, FP, TN, and FN represent the numbers of true positives, false positives, true negatives, and false negatives, respectively. All metrics had values ranging between 0 and 1, with 1 being the optimum.

To determine the optimal classification threshold for calculating sensitivity, specificity, F1-score, and the confusion matrix, we used Youden’s J-index (defined as sensitivity + specificity – 1) as the criterion. The threshold corresponding to the maximum J-index on the ROC curve was selected for each model. All threshold-dependent metrics reported in the study were computed based on this value, ensuring a balanced trade-off between sensitivity and specificity.

### Statistical analysis

We conducted a statistical analysis to compare the patients with and without severe exacerbations during the 1-year follow-up periods. Additionally, we evaluated the relationship between each feature and the dependent variable (severe exacerbation). Continuous variables were summarized using means and standard deviations, while categorical variables were represented as counts and proportions. Continuous variables were summarized using means and standard deviations, while categorical variables were represented as counts and proportions. To ensure the appropriateness of statistical methods, we assessed the normality of continuous variables using the Shapiro-Wilk test and homogeneity of variance using Levene’s test. Based on these results, we compared continuous variables between groups using the Student’s t-test for normally distributed data and the Mann-Whitney U test for non-normally distributed data. Categorical features were analyzed using the chi-square (χ2) test or Fisher’s exact test, as appropriate. Statistical significance was set at P-values < 0.05. All statistical analyses were performed using the R statistical software version 4.1.2 (R Core Team 2021).

## Supplementary Information

Below is the link to the electronic supplementary material.

Supplementary Material 1

## Data Availability

The data supporting this study’s findings are available from the corresponding author upon reasonable request.

## Code availability

The code used in this study is available from the corresponding author upon reasonable request.
