# Supplementary material for: AI based prediction of severe exacerbation in Asian bronchiectasis patients using the KMBARC registry
Source: Sci Rep. 2026 Feb 25;16:11017. doi: 10.1038/s41598-026-38968-9 (PMC13043856; doi:10.1038/s41598-026-38968-9)
Supplement: Supplementary file 1 — Supplementary Material 1 [file 41598_2026_38968_MOESM1_ESM.pdf]

Supplementary Table S1. Hyperparameters of each model used for severe AE prediction.

Model	Hyperparameters	values
**XGBoost**	n_estimators	100
	reg_alpha	0·1
	reg_lambda	0·1
	subsample	1·0
	colsample_bytree	0·8
	gamma	1
	learning_rate	0·1
	max_depth	3
	min_child_weight	1
**LR**	C	0·1
	penalty	L1
**MLP**	number of hidden nodes	[32, 64, 128]
	dropout rate	0·2
	optimizer	Adam
	learning rate	1.0e-3
	batch size	8
	loss function	weighted binary cross-entropy

Abbreviations: XGBoost, extreme gradient boosting; LR, logistic regression; MLP, multilayer perceptron.

Supplementary Figure S1. Architecture of the MLP model for predicting severe AE probability.

Abbreviations: MLP, multilayer perceptron, AE, acute exacerbation.

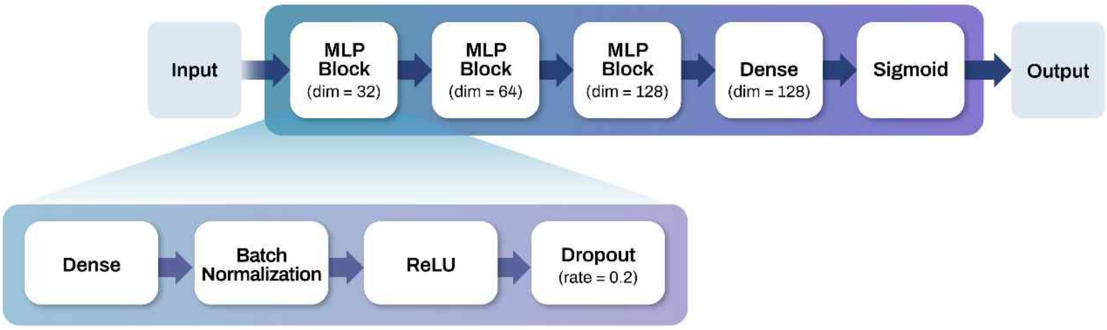

Supplementary Figure S2. Precision–recall (PR) curves of Extreme Gradient Boosting (XGBoost), Logistic Regression (LR), and Multilayer Perceptron (MLP) for predicting severe acute exacerbation (AE) in patients with bronchiectasis.

Abbreviations: XGBoost, Extreme gradient boosting; LR, logistic regression; MLP, multilayer perceptron.

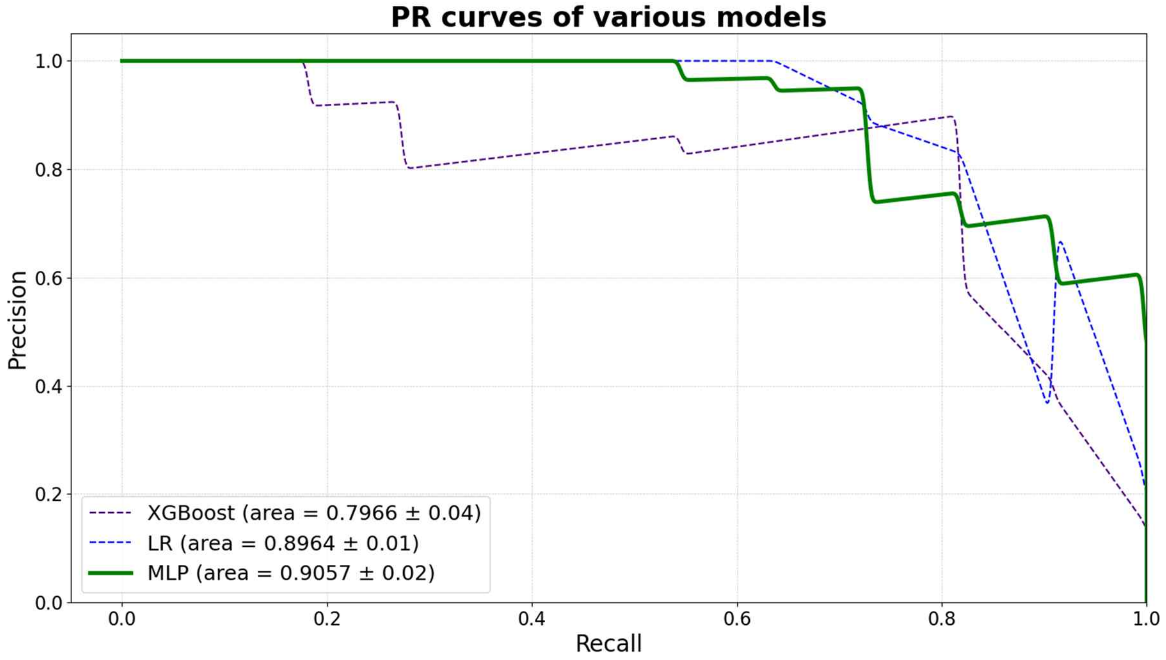

Supplementary Figure S3. Shapley Additive Explanation (SHAP) analyses for Extreme Gradient Boosting (XGBoost) and Logistic Regression (LR) used to estimate the risk of severe acute exacerbation (AE). Subfigures (a) and (b) show the SHAP summary and importance plots for XGBoost, respectively, while (c) and (d) present the corresponding plots for LR.

Abbreviations: BSI, *Bronchiectasis* Severity Index; LAMA, long-acting muscarinic antagonist; LABA, long-acting beta-2 agonist; COPD,Chronic Obstructive Pulmonary Disease; ICS, inhaled corticosteroid; OCS, Oral corticosteroid.

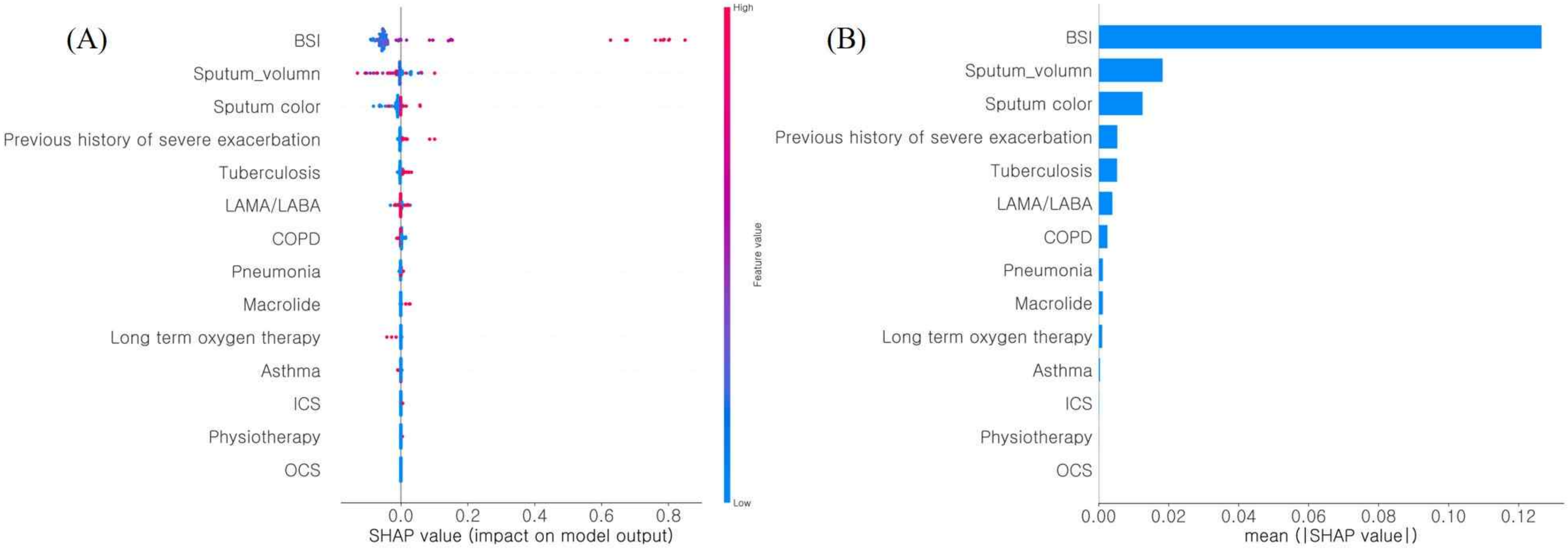

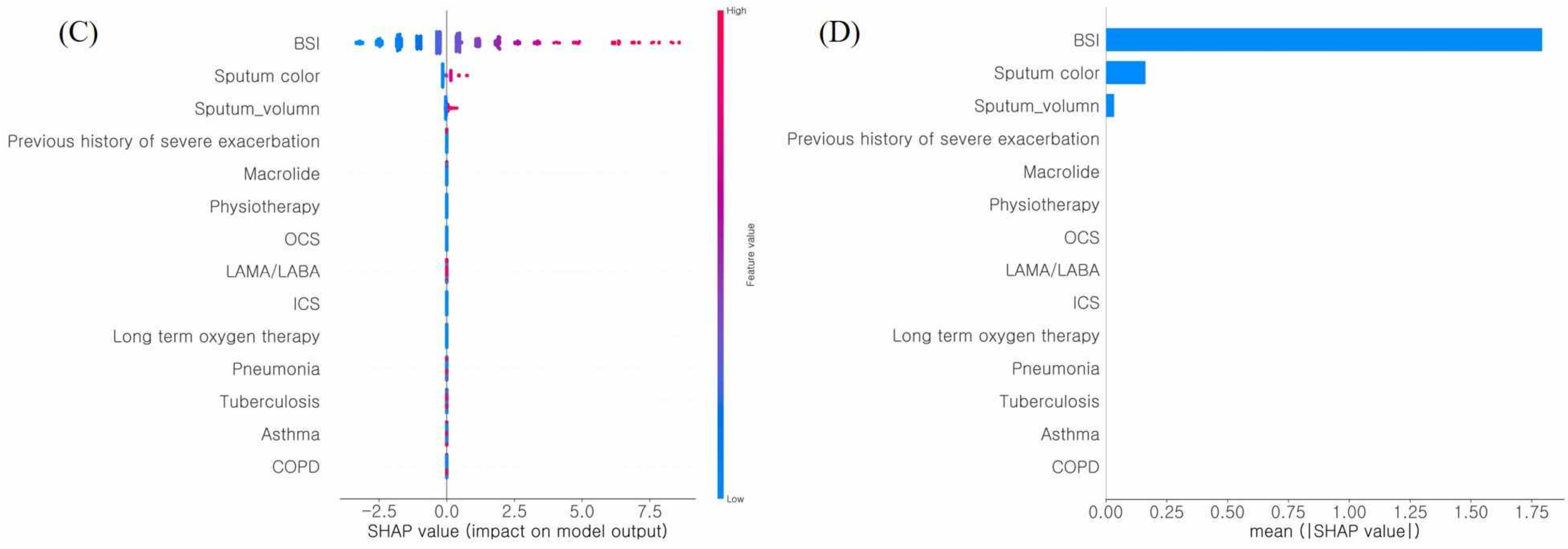
(C)
BSI
Sputum color
Sputum_volumn
Previous history of severe exacerbation
Macrolide
Physiotherapy
OCS
LAMA/LABA
ICS
Long term oxygen therapy
Pneumonia
Tuberculosis
Asthma
COPD
−2.5 0.0 2.5 5.0 7.5
SHAP value (impact on model output)
High
Feature value
Low
(D)
BSI
Sputum color
Sputum_volumn
Previous history of severe exacerbation
Macrolide
Physiotherapy
OCS
LAMA/LABA
ICS
Long term oxygen therapy
Pneumonia
Tuberculosis
Asthma
COPD
0.00 0.25 0.50 0.75 1.00 1.25 1.50 1.75
mean (|SHAP value|)
